# ChIP-Chip Designs to Interrogate the Genome of *Xenopus* Embryos for Transcription Factor Binding and Epigenetic Regulation

**DOI:** 10.1371/journal.pone.0008820

**Published:** 2010-01-21

**Authors:** Robert C. Akkers, Simon J. van Heeringen, J. Robert Manak, Roland D. Green, Hendrik G. Stunnenberg, Gert Jan C. Veenstra

**Affiliations:** 1 Department of Molecular Biology, Faculty of Science, Radboud University Nijmegen, Nijmegen, The Netherlands; 2 Roche Nimblegen, Inc., Madison Wisconsin, United States of America; 3 Department of Biology, University of Iowa, Iowa City, Iowa, United States of America; University of Birmingham, United Kingdom

## Abstract

**Background:**

Chromatin immunoprecipitation combined with genome tile path microarrays or deep sequencing can be used to study genome-wide epigenetic profiles and the transcription factor binding repertoire. Although well studied in a variety of cell lines, these genome-wide profiles have so far been little explored in vertebrate embryos.

**Principal Findings:**

Here we report on two genome tile path ChIP-chip designs for interrogating the *Xenopus tropicalis* genome. In particular, a whole-genome microarray design was used to identify active promoters by close proximity to histone H3 lysine 4 trimethylation. A second microarray design features these experimentally derived promoter regions in addition to currently annotated 5′ ends of genes. These regions truly represent promoters as shown by binding of TBP, a key transcription initiation factor.

**Conclusions:**

A whole-genome and a promoter tile path microarray design was developed. Both designs can be used to study epigenetic phenomena and transcription factor binding in developing *Xenopus* embryos.

## Introduction

Chromatin immunoprecipitation combined with either microarray hybridization (ChIP-chip) or sequencing (ChIP-seq) allows to determine genomic association of DNA binding proteins and to analyze epigenetic regulation [1], [2], [3]. Active transcription coincides with epigenetic features like the presence of the histone H3 lysine 4 trimethylation (H3K4me3) and acetylation of lysine 9 of histone H3 (H3K9ac) at the 5′ end of transcriptionally active genes [4], [5], [6]. In the context of development little is known about epigenetic marks in embryos using ChIP profiling. H3K4me3 ChIP-chip using zebrafish embryos identified actively transcribed embryonic genes [7]. The latter mark was also detected in *Drosophila* embryos at promoter regions [8]. ChIP sequencing of H3K4me3 and H3K27me3, an inactive histone mark, was used to enhance 5′ gene annotation in *Xenopus* and to analyze spatial regulation of gene expression during gastrulation [9]. The two marks only appear after the onset of transcription at the mid-blastula transition with a hierarchy in deposition of H3K4me3 and H3K27me3, respectively.

The challenge in the near future is to elucidate epigenetic transitions and transcription networks that underlie early vertebrate development, the analysis of which will be facilitated by genome binding site analyses using either ChIP-chip or ChIP-seq. Genome tiling arrays are most cost-efficient if a dedicated design covering the relevant regulatory regions is used. Such a design can be made on the basis of annotation, or alternatively using experimental data of histone modifications like H3K4me3 that identify these regulatory regions. Here we present results obtained using a five array whole-genome tiling *X. tropicalis* design and a promoter microarray design based on chromatin decorated with H3K4me3. The data show that both microarray designs are suitable for studies of histone modifications and transcription factor binding events in early *Xenopus* embryogenesis.

## Results

### Genome-Wide ChIP-Chip Using *X. tropicalis* Gastrula Embryos

**Figure 1:**
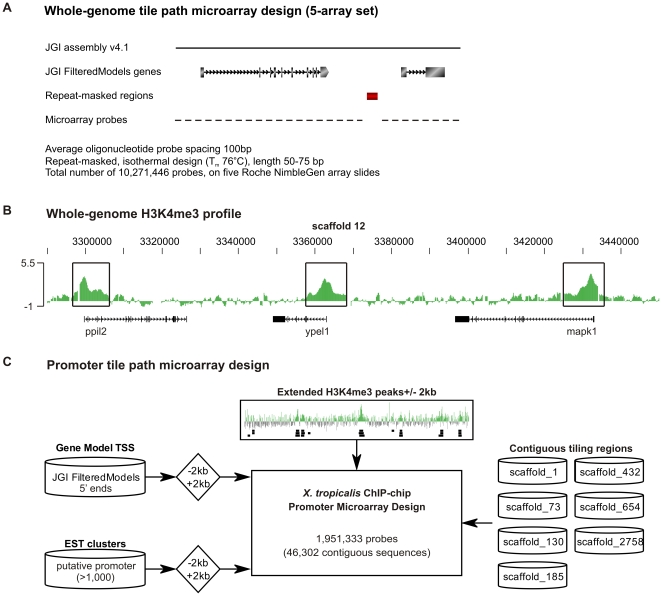
Whole-genome H3K4me3 ChIP-chip of gastrula embryos. (**A**) Overview of the whole-genome tile path microarray design. (**B**) H3K4me3 profile for peptidylprolyl isomerase cyclophilin-like 2 (*ppil2*), yippee-like 1 (*ypel1*) and mitogen-activated protein kinase 1 (*mapk1*) that are enriched with H3K4me3 signals at the 5′ end visualized using the UCSC Genome Browser. (**C**) Overview of the promoter tile path microarray design.

To explore epigenetic features of gastrula-stage *Xenopus tropicalis* embryos, chromatin was harvested (Nieuwkoop-Faber stage 11–12) and immunoprecipitated using H3K4me3 antibodies. The purified DNA of H3K4me3-ChIP was amplified using a T7 amplification protocol [10] and hybridized to a newly designed Nimblegen whole-genome tilepath set of five microarrays covering the complete *X. tropicalis* genome (Joint Genome Institute v4.1). The microarrays used each consist of 2.1M isothermal (76°C) probes of 50–75bp in length that are spaced by an average of 100 bp in the genome, with the exclusion of repeat-masked regions (Figure 1A). The H3K4me3 signal was present at the 5′ end of genes, as is shown for a typical euchromatic genomic region, covering the genes *ppil2*, *ypel1* and *mapk1* (Figure 1B). To generate a single promoter microarray design (Figure 1C), all putative H3K4me3 positive regions (27,019 regions, see material & methods for details), together with the 5′ ends of all 27,916 Joint Genome Institute FilteredModels genes (−2 kb/+2 kb) were selected. In addition, approximately 1,000 regions were included based on clusters of 5′ ends of ESTs (−2 kb/+2 kb). In total, the promoter microarray design contains 1,951,339 probes, comprised of 46,302 contiguous sequences with a median length of ∼4 kb. In addition, to measure genomic background ChIP-chip signals for the purpose of peak detection the contiguous sequence of several scaffolds were included completely.

### Promoter Microarray H3K4me3 ChIP-Chip

**Figure 2:**
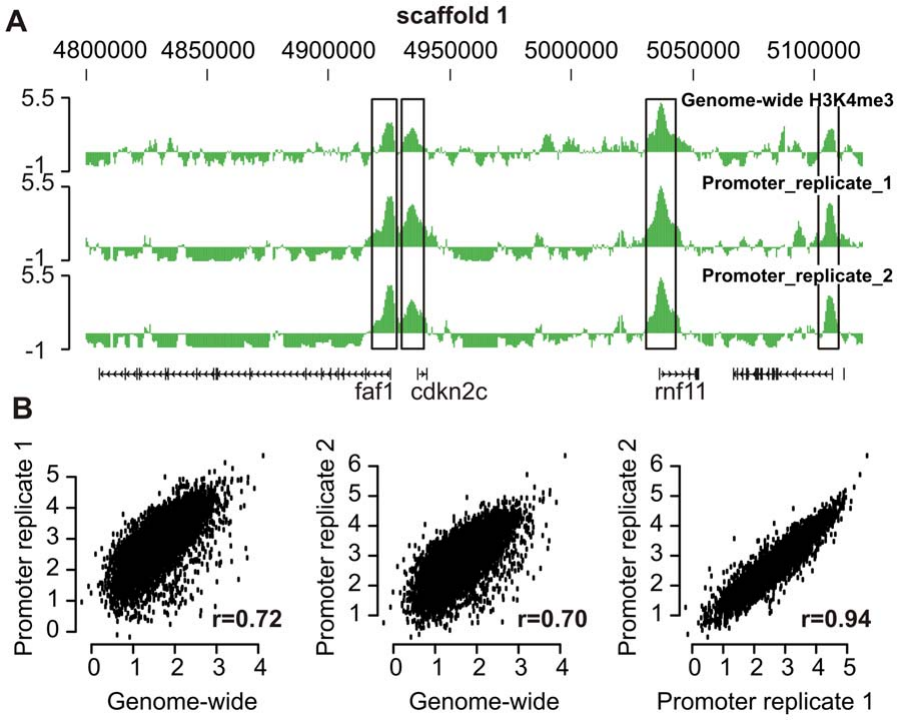
Promoter microarray H3K4me3 ChIP-chip. (**A**) Part of scaffold_1 is visualized using the UCSC Genome Browser. The four H3K4me3 enriched regions; Fas associated factor 1 (*faf1*), cyclin-dependent kinase inhibitor 2C (*cdkn2c*), ring finger protein 11 (*rnf11*) and an unknown protein are preserved in the three independent experiments. (**B**) Correlation of the genome-wide tiling and promoter microarray design ChIP-chip experiments; Pearson correlation r = 0.72, 0.70 and 0.94 respectively (p<2.2 * 10^−16^).

Two new H3K4me3 gastrula ChIP-chip samples were generated, amplified using the T7 method and hybridized to the new promoter microarray design. The two biological replicate samples are quite similar in terms of location and shape of the enriched regions as is shown for part of scaffold 1 (Figure 2A). We determined the correlation between the genome-wide ChIP-chip and the promoter ChIP-chip experiments (Figure 2B). The mean signal per peak of the genome-wide experiment correlated with the experiments using the promoter microarray design (r = 0.72 and r = 0.70 respectively, p<2.2 * 10^−16^). The correlation between the promoter microarray experiments was r = 0.94 (p<2.2 * 10^−16^). This shows that ChIP-chip experiments based on genome-wide tiling or a promoter microarray design are highly reproducible and that the biological variance between different batches of embryos is relatively low.

### Analyses of H3K4me3 Triplicate

**Figure 3:**
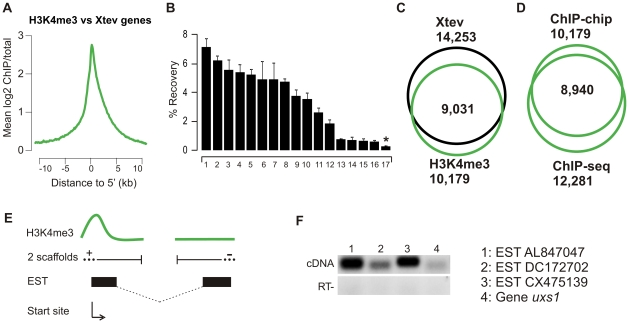
Analyses of H3K4me3 triplicate. (**A**) Distributions of H3K4me3 at annotated Xtev genes. (**B**) ChIP-qPCR validation of randomly selected enriched H3K4me3 regions on three new generated biological replicates. 16 out of 17 of H3K4me3 enriched regions are validated as true positives with an average enrichment >2.5-fold over background (genomic locus on scaffold_1:6458583-6458633). Asterisk indicates a locus with <2.5-fold enrichment (experimental FDR <0.06). Error bars represent the SEM of three independent ChIP experiments. (**C**) Overlap of H3K4me3-enriched regions and Xtev genes (p<10^−25^). (**D**) Overlap of H3K4me3 ChIP-chip and ChIP-seq peaks. Of the peaks found with ChIP-chip, 88% are also detected with ChIP-seq [9] (p<10^−25^). (**E**) ‘Orphan’ H3K4me3 peaks linking different scaffolds. Schematic overview of orphan linkage of two scaffolds using H3K4me3 signals and the EST database. The start site is determined by the H3K4me3 peak and the transcription unit combined with the EST information of the other scaffold. (**F**) RT-PCR validation of ESTs linking two scaffolds using primers that align to two different scaffolds: scaffold 649 and 145 (EST AL847047), scaffold 675 and 1278 (EST DC172702), scaffold 716 and 1312 (EST CX475139) and scaffold 1393 and 307 (uxs1). cDNA used for validation was generated from RNA isolated from stage 11–12 embryos.

We determined the profile of the H3K4me3 signal over the 5′ ends of *X. tropicalis* experimentally validated genes (Xtev [9]). H3K4me3 is predominantly found at the transcription start site (TSS; Figure 3A) as expected. The distribution of the H3K4me3 mark over Joint Genome Institute FilteredModels genes shows a similar profile (Figure S1). We determined the number of H3K4me3-enriched regions using TileMap [11]. In total 10,179 H3K4me3 positive regions were detected. To validate this peak set, randomly selected targets were tested in ChIP-qPCR using three new biological ChIP samples (Figure 3B). Using two negative loci on scaffold 1 (scaffold_1:6458583-6458633) and scaffold 919 (scaffold_919:126357-126407) to determine the genomic background, 16 out of 17 regions were enriched more than 2.5-fold (Figure S2, FDR <0.06). The H3K4me3 peak set shows a high degree of overlap with 5′ ends of Xtev genes (within 1 kb of the TSS, p<10^−25^; Figure 3C). Up to 89% of the H3K4me3-enriched regions (9,031 of 10,179) are within 1kb of genes. A list of all H3K4me3-enriched regions and associated Xtev, JGI FilteredModels and RefSeq genes is supplied as a supplemental table (Table S1). The peaks of genome-wide ChIP-chip were compared to ChIP-seq of H3K4me3 of gastrula-stage embryos [9]. 88% of the peaks determined by ChIP-chip are also detected by ChIP-seq (p<10^−25^; Figure 3D). These results show that H3K4me3-based ChIP-chip experiments are highly reproducible and that both experimental platforms identify an almost identical collection of H3K4me3-enriched genomic sequences. Moreover, integration of ChIP and EST data allows linking of ‘orphan’ H3K4me3 peaks to gene models on different genomic scaffolds in cases where promoter and coding regions were placed on different sequence contigs during genome assembly (schematic overview, Figure 3E). Both 5′ and 3′ exons can be located on different scaffolds. To test this, using the EST information, RT-PCR primers were designed that align to different scaffolds to amplify gastrula *X. tropicalis* cDNA. Four random chosen examples were validated by this approach as transcription units that go together but are assembled to different scaffolds (Figure 3F). Importantly, genuine 5′ ends of genes can be identified by their enrichment for H3K4me3, even if the 5′ end and the gene body are located on different scaffolds. In total, for 991 loci we detected H3K4me3-enrichment and EST annotation to two scaffolds (Table S2). We also tested the enrichment for H3K9ac for a limited number of genomic regions and found that 34 out of 43 (79%) H3K4me3 regions are also enriched for H3K9ac (data not shown). These results show that the genome-wide ChIP-chip design is a useful tool to interrogate gene regulatory regions in *Xenopus*.

### TBP-Enriched Regions

**Figure 4:**
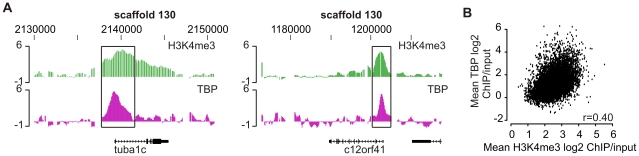
TBP-enrichment at loci of promoter microarray design. (**A**) Independent genes visualized in the UCSC Genome Browser for H3K4me3 (green) and TBP (pink). Left panel represents tubulin, alpha 1c (*tuba1c*) and the right panel human chromosome 12 open reading frame 41 (*c12orf41*). (**B**) Correlation of H3K4me3 ChIP-chip and TBP promoter ChIP-chip. Spearman rank r = 0.40 (p<2.2 * 10^−16^).

To examine the application value of our promoter microarray design for transcription factors we studied TATA-binding protein (TBP), a key initiation factor that is known to bind to promoter regions. T7 amplified TBP ChIP and input DNA was hybridized to the promoter microarray. Many TBP-enriched regions colocalize with H3K4me3 enriched regions as is seen for example for tubulin alpha 1c (Figure 4A; left panel). TBP is also found at H3K4me3-positive regions that lack gene annotation. For example, the promoter region of an open reading frame with no known function was bound by TBP in the presence of H3K4me3 (Figure 4A; right panel). We determined the correlation of H3K4me3 peak regions and TBP to reveal the relation between epigenetic marks and transcription initiation factors (Figure 4B). Although correlation is less than observed for the H3K4me3 replicates, many H3K4me3-positive loci are bound by TBP and the correlation is highly significant (r = 0.40, p<2.2 * 10^-16^).

## Discussion

Our results present ChIP microarray designs for *X. tropicalis*. So far gene regulatory networks and epigenetic regulation during vertebrate embryogenesis have not extensively been studied using genome-wide binding analysis. By contrast, many epigenetic profiles have been established for human cells in culture [12], providing a solid conceptual framework to analyze epigenetic regulation in other systems. The presence of H3K4me3 at the 5′ end of transcribed genes is consistent in many experimental systems [5], [7], [8], [9], [13], [14], [15], [16], [17], [18].

The number of H3K4me3-enriched regions in *Xenopus* gastrula embryos based on ChIP-chip or ChIP-seq [9] is comparable (88% overlap). For *Xenopus*, a promoter microarray design based on current Joint Genome Institute gene annotation would lose valuable information regarding the TSS, since a large number of H3K4me3-enriched regions do not overlap with current JGI gene annotation within 1 kb (40%; 4,116 out of 10,179). An experimental approach to the design of a promoter microarray, based on H3K4me3-enriched regions, is therefore highly favored. Although many promoters featured on this microarray design are expected to be active in multiple tissues and stages of development, it should be noted that most comprehensive results will be obtained with this design when analyzing promoter binding events in gastrula stage embryos.

The biological variation of the H3K4me3 modification is quite low in *Xenopus* gastrula embryos. We also performed TBP ChIP-chip and find TBP peaks overlapping with H3K4me3 peaks, as expected for active promoters. The correlation between H3K4me3 and TBP, though highly significant, is not as strong as that observed between biological replicates of H3K4me3 enrichment. However, it should be noted that TBP paralogs have been identified that are required for transcription initiation during embryogenesis [19], [20], [21] and some promoters decorated with H3K4me3 do not recruit TBP. It will be worthwhile to study the dynamics of TBP related factors during early *Xenopus* development.

This work describes microarray designs which are made available to the community, thereby facilitating future studies in *Xenopus* embryogenesis using ChIP-chip. For the analysis of promoter binding the promoter microarray design for *X. tropicalis* can be used. For a genome-wide analysis of DNA binding or epigenetic regulation the five microarray set can be used. These types of genomic studies will enhance our understanding of transcriptional networks and developmental pathways and identify novel targets genes important for vertebrate development.

## Materials and Methods

### Chromatin Immunoprecipitation

Animal work has been conducted according to relevant national and international guidelines and following approval of the institutional review board for animal experimentation (Dierexperimenten commissie). *Xenopus tropicalis* embryos were obtained from a natural mating procedure after human chorionic gonadotropin injection, dejellied in 2% cysteine and collected at the indicated stage. Chromatin harvesting from 300 gastrula *X. tropicalis* embryos and ChIP (15 embryo equivalents) was performed as described [20] with two minor modifications: 12.5 µl of Prot A/G beads (Santa Cruz) were used and during reversal of the crosslinking proteinase K was omitted from the buffer. We used α-H3K4me3 (Abcam), α-H3K9ac (Upstate) and α-TBP (SL33) antibodies.

### RNA Isolation and cDNA Synthesis

Total RNA was isolated from 20 gastrula *X. tropicalis* embryos using TRIzol reagent (Invitrogen). 5 µg of RNA was subjected to a reverse transcription reaction in the presence (cDNA) or absence (RT-) of SuperScriptIII (Invitrogen).

### Quantitative PCR

5 µl of ChIP material (0.375 embryo equivalents) or 10 times diluted cDNA or RT- was used for quantitative PCR. PCR reactions were performed on a MyIQ single color real-time PCR detection system (BioRad) using iQ SYBR Green Supermix (BioRad). Primer sequences are available in Table S3.

### T7 Amplification

T7 amplification procedure was essentially identical as described by [10]. In short, after removal of the 5′ phosphate groups the DNA fragments enzymatically acquired a T-tail followed by T7 promoter incorporation. *In vitro* transcription coupled to 1^st^ and 2^nd^ strand synthesis was done to acquire dsDNA.

### ChIP-Chip

ChIP-chip samples were prepared according to the manufacturers protocol (Roche Nimblegen). In short, DNA yielded by 4 ChIP reactions (60 embryo equivalents) was pooled and amplified using the T7 strategy. DNA purified from de-crosslinked chromatin (i.e. input, genomic control) was also amplified. Per labeling reaction 4 µg DNA was required (post-amplification). ChIP experimental samples were labeled with Cy3 and genomic control samples with Cy5. Samples were hybridized to the NimbleGen ChIP-chip microarrays by Research and Development at Roche NimbleGen (Madison, WI, USA).

### Promoter Microarray Design

H3K4me3-positive regions to include on the promoter microarray design were selected based on relaxed criteria, to include as many putative positive regions while still keeping to the practical size limit of one HD2 Nimblegen microarray (∼2.1 million probes). A relatively simple procedure was chosen in order to retrieve an inclusive peak set. This peak selection was only used to design the promoter microarray, not for any further comparisons. Peak detection was performed using a sliding window operation, with three different thresholds for three window lengths (3, 4 or 5 consecutive probes). These different windows were used to select relatively small high peaks, as well as somewhat broader, lower peaks. As a control, all probes were randomized per microarray and sliding window detection on the randomized probes was performed. On both real and randomized data the sliding window detection was repeated with an increasing threshold. A definitive threshold was chosen such that the number of peaks in the randomized set divided by the number of peaks in the real data, the theoretical FDR, was <10%. All overlapping positive regions were merged and each region was extended both up- and downstream by 2 kb. In total this resulted in 27,019 putative promoter regions, the same order of magnitude as the annotated genes (27,916 JGI FilteredModels genes). The design was extended by the addition of several (partially overlapping) datasets. All 5′ gene ends of JGI FilteredModels genes were added, as well a set of putative transcription start sites based on EST evidence (SJvH, W.Akhtar, RCA and GJCV, manuscript in preparation), both extended by 2 kb up- and downstream. Finally, the complete tile path of scaffold_1, scaffold_73, scaffold_130, scaffold_185, scaffold_432, scaffold_654 and scaffold_2758 was included. These regions can be used to assess genomic background in peak calling applications. The complete design consists of 1,951,339 probes, which fits on a single 2.1M Nimblegen microarray, covering a total of 46,302 contiguous sequences with a median length of ∼4 kb.

### Peak Detection

To call H3K4me3 peaks based on the three biological replicates (one genome-wide and two replicates on the promoter microarray design), TileMap [11] was chosen. In contrast to the selection of positive regions for the promoter microarray design, where an inclusive set was preferred, in this case the aim was to provide a set of high-confidence H3K4me3 peaks. As Tilemap is based on a statistical model that incorporates the replicate data, it is a more solid approach than a simple sliding window procedure. Before peak detection all probes matching more than one time to the *X. tropicalis* genome (JGI 4.1) were removed. The following Tilemap parameters were set: method HMM, posterior probability 0.01, expected hybridization length 10. All other parameters were left to default settings. All resulting peaks of only one probe were removed, and all peaks within 1.5 kb were merged. The peaks for TBP (9,558) were called using Tilemap with default parameters. The files containing the H3K4me3 peaks, the TBP peaks, the number of matches for each probe to *X. tropicalis* JGI 4.1, as well as the Tilemap parameter files are available as supplementary information through GEO Series GSE19413.

### Orphan H3K4me3 Linkage

All mapped *X. tropicalis* ESTs were downloaded from the UCSC Genome Browser (xenTro2, 12-2007). ESTs were filtered based on two criteria: 1) mapping to exactly two scaffolds and 2) a H3K4me3 peak within 1 kb of the EST on one of the scaffolds. All unique combinations of two scaffolds with evidence of more than one filtered EST were kept and are summarized in Table S2.

### Comparisons between ChIP-Chip Replicates and between ChIP-Chip and ChIP-Seq Peaks

To calculate the correlation of the genome-wide replicate to the two replicates on the promoter microarray the mean per-peak log2 ChIP/input signal was calculated per replicate for all the 10,179 peaks. The correlation coefficient between these replicate per-peak signals was calculated using the Pearson's correlation. To calculate the overlap between H3K4me3 determined by ChIP-chip and ChIP-seq, the H3K4me3 ChIP-seq peaks determined previously (GSM352202_H3K4me3_enriched.bed, available through GEO Series GSE14025) [http://www.ncbi.nlm.nih.gov/geo/query/acc.cgi?acc=GSE14025] were intersected with the peaks determined by ChIP-chip in this study. All ChIP-seq peaks overlapping with at least 1bp were counted as detected by both methods.

### Data Availability and Supplemental Data

The data and the microarray designs used to generate the data have been deposited in NCBI's Gene Expression Omnibus [22] and are accessible through GEO Series accession number GSE19413 (http://www.ncbi.nlm.nih.gov/geo/query/acc.cgi?acc=GSE19413). Improved versions (v2) of the *X. tropicalis* genome-wide microarray design and the promoter microarray design are available at the authors' web site (http://www.ncmls.nl/gertjanveenstra). These updated designs include extra control probes (random controls and probes corresponding to Arabidopsis BAC clones F19K16, accession AC011717, and F24B22, accession AL132957). The Arabidopsis sequences can be used as spike-in controls. In addition, all scaffolds of the whole-genome tiling microarray design were randomly distributed over the 5 microarrays of the array set to prevent that any single microarray features all the small scaffolds. The small (high number) scaffolds contain significantly fewer genes and distributing these scaffolds evenly over the 5-array set may prevent signal normalization issues. Arrays featuring these designs can be purchased from Roche Nimblegen.

## Supporting Information

Table S1 H3K4me3-enriched regions and annotation. List of all H3K4me3-enriched regions and associated annotation (within 1 kb) for Xtev, JGI FilteredModel and RefSeq genes.
(1.36 MB XLS)

Table S2 Orphan H3K4me3 peaks: 991 regions are enriched for H3K4me3 and have EST annotation on two scaffolds.
(0.11 MB XLS)

Table S3 Primer sequences.
(0.02 MB XLS)

Figure S1 Distribution of H3K4me3 enrichment at JGI FilteredModels genes.
(0.20 MB TIF)

Figure S2 ChIP-qPCR validation of randomly selected H3K4me3-enriched regions. (A) Average enrichment (three experiments) determined for a genomic locus on scaffold 1 (scaffold_1:6458583-6458633). Asterisk indicates enrichment less than 2.5. (B) Average enrichment relative to a genomic locus on scaffold 919 (scaffold_919:126357-126407). Error bars represent the SEM of three biological replicates.
(0.61 MB TIF)

## References

[1] Bennett S (2004). Solexa Ltd.. Pharmacogenomics.

[2] Mendenhall EM, Bernstein BE (2008). Chromatin state maps: new technologies, new insights.. Curr Opin Genet Dev.

[3] Ren B, Robert F, Wyrick JJ, Aparicio O, Jennings EG (2000). Genome-wide location and function of DNA binding proteins.. Science.

[4] Berger SL (2002). Histone modifications in transcriptional regulation.. Curr Opin Genet Dev.

[5] Bernstein BE, Kamal M, Lindblad-Toh K, Bekiranov S, Bailey DK (2005). Genomic maps and comparative analysis of histone modifications in human and mouse.. Cell.

[6] Santos-Rosa H, Schneider R, Bannister AJ, Sherriff J, Bernstein BE (2002). Active genes are tri-methylated at K4 of histone H3.. Nature.

[7] Wardle FC, Odom DT, Bell GW, Yuan B, Danford TW (2006). Zebrafish promoter microarrays identify actively transcribed embryonic genes.. Genome Biol.

[8] Schuettengruber B, Ganapathi M, Leblanc B, Portoso M, Jaschek R (2009). Functional anatomy of polycomb and trithorax chromatin landscapes in Drosophila embryos.. PLoS Biol.

[9] Akkers RC, van Heeringen SJ, Jacobi UG, Janssen-Megens EM, Francoijs KJ (2009). A hierarchy of H3K4me3 and H3K27me3 acquisition in spatial gene regulation in Xenopus embryos.. Dev Cell.

[10] Liu CL, Schreiber SL, Bernstein BE (2003). Development and validation of a T7 based linear amplification for genomic DNA.. BMC Genomics.

[11] Ji H, Wong WH (2005). TileMap: create chromosomal map of tiling array hybridizations.. Bioinformatics.

[12] Barski A, Cuddapah S, Cui K, Roh TY, Schones DE (2007). High-resolution profiling of histone methylations in the human genome.. Cell.

[13] Bernstein BE, Mikkelsen TS, Xie X, Kamal M, Huebert DJ (2006). A bivalent chromatin structure marks key developmental genes in embryonic stem cells.. Cell.

[14] Mikkelsen TS, Ku M, Jaffe DB, Issac B, Lieberman E (2007). Genome-wide maps of chromatin state in pluripotent and lineage-committed cells.. Nature.

[15] Pan G, Tian S, Nie J, Yang C, Ruotti V (2007). Whole-genome analysis of histone H3 lysine 4 and lysine 27 methylation in human embryonic stem cells.. Cell Stem Cell.

[16] Roh TY, Zhao K (2008). High-resolution, genome-wide mapping of chromatin modifications by GMAT.. Methods Mol Biol.

[17] Wang Z, Zang C, Rosenfeld JA, Schones DE, Barski A (2008). Combinatorial patterns of histone acetylations and methylations in the human genome.. Nat Genet.

[18] Zhao XD, Han X, Chew JL, Liu J, Chiu KP (2007). Whole-genome mapping of histone H3 Lys4 and 27 trimethylations reveals distinct genomic compartments in human embryonic stem cells.. Cell Stem Cell.

[19] Jacobi UG, Akkers RC, Pierson ES, Weeks DL, Dagle JM (2007). TBP paralogs accommodate metazoan- and vertebrate-specific developmental gene regulation.. Embo J.

[20] Jallow Z, Jacobi UG, Weeks DL, Dawid IB, Veenstra GJ (2004). Specialized and redundant roles of TBP and a vertebrate-specific TBP paralog in embryonic gene regulation in Xenopus.. Proc Natl Acad Sci U S A.

[21] Veenstra GJ, Weeks DL, Wolffe AP (2000). Distinct roles for TBP and TBP-like factor in early embryonic gene transcription in Xenopus.. Science.

[22] Edgar R, Domrachev M, Lash AE (2002). Gene Expression Omnibus: NCBI gene expression and hybridization array data repository.. Nucleic Acids Res.

