# Supplementary figures and images for: ChIP-Chip Designs to Interrogate the Genome of Xenopus Embryos for Transcription Factor Binding and Epigenetic Regulation
Source: PLoS One. 2010 Jan 21;5(1):e8820. doi: 10.1371/journal.pone.0008820 (PMC2809088; doi:10.1371/journal.pone.0008820)

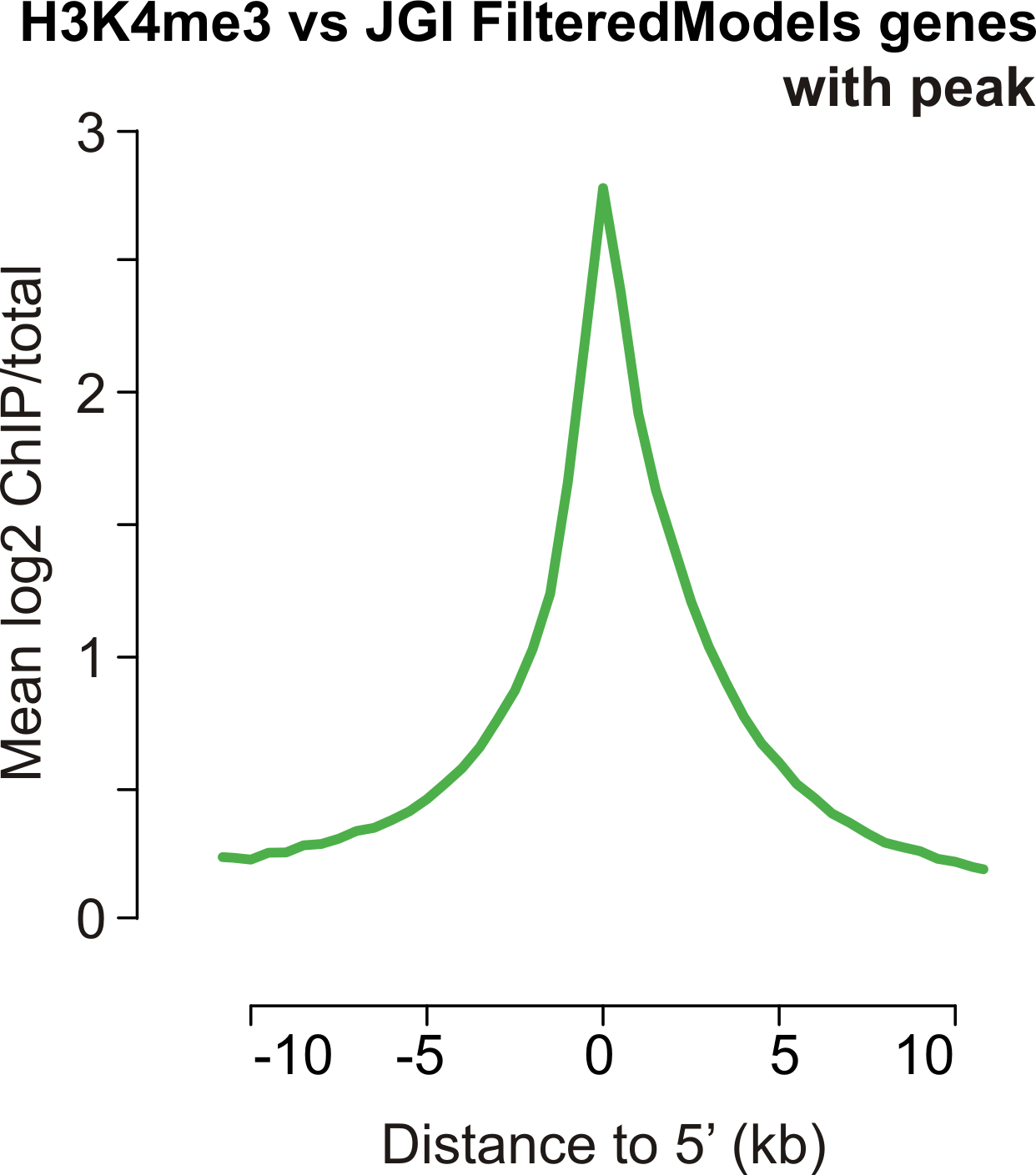

Supplement: Figure S1 — Distribution of H3K4me3 enrichment at JGI FilteredModels genes. (0.20 MB TIF) [file pone.0008820.s004.tif]

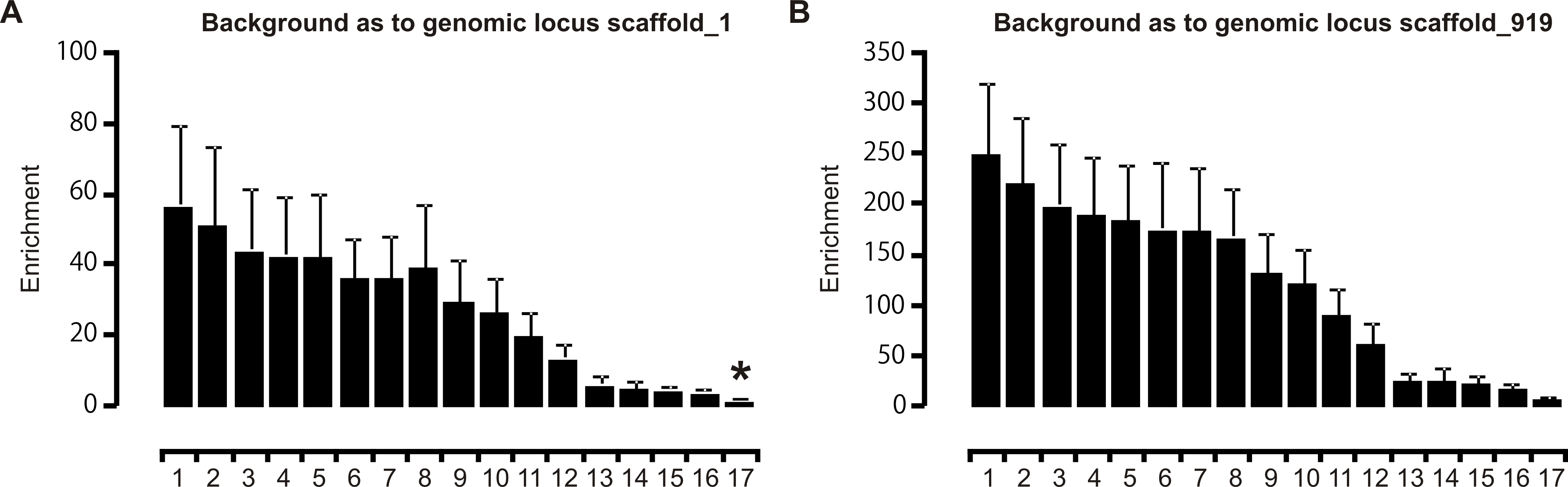

Supplement: Figure S2 — ChIP-qPCR validation of randomly selected H3K4me3-enriched regions. (A) Average enrichment (three experiments) determined for a genomic locus on scaffold 1 (scaffold_1:6458583-6458633). Asterisk indicates enrichment less than 2.5. (B) Average enrichment relative to a genomic locus on scaffold 919 (scaffold_919:126357-126407). Error bars represent the SEM of three biological replicates. (0.61 MB TIF) [file pone.0008820.s005.tif]
